# First report of canine morbillivirus infection of adipose tissue-derived stem cells from dogs with distemper

**DOI:** 10.14202/vetworld.2022.1835-1842

**Published:** 2022-07-27

**Authors:** Fabiola Altamirano-Samaniego, Javier Enciso-Benavides, Nancy Rojas, Juan Manuel Iglesias-Pedraz, Nathaly Enciso, Matia Fossatti, Javier Enciso

**Affiliations:** 1 Grupo de Medicina Regenerativa, Universidad Científica del Sur, Lima, Perú; 2 Laboratorio de Células Madre, Clínica Veterinaria Enciso, Lima, Perú; 3 Laboratorio de Microscopía Electrónica, Facultad de Medicina, Universidad Nacional Mayor de San Marcos, Lima, Perú; 4 Laboratorio de Bioquímica y Genética Molecular, Universidad Científica del Sur, Lima, Perú

**Keywords:** adipose stem cells, canine distemper disease, canine morbillivirus, cell therapy, viral latency

## Abstract

**Background and Aim:**

Ribonucleic acid viruses remain latent in different cell types, including mesenchymal stem cells; however, the distemper virus remains undetected in these cells. This study aimed to determine whether adipose stem cells (ASCs) from dogs with distemper disease are infected with the canine morbillivirus (CM).

**Materials and Methods:**

Twelve dogs with the neurological phase of the disease and who were positive for CM by reverse transcription polymerase chain reaction (RT-PCR), were studied. ASCs from adipose tissue of the lesser omentum of these infected dogs were isolated and characterized. Direct fluorescence was used to detect the viral antigen in cell cultures. Flow cytometry and RT-PCR identified detectable quantities of the virus in two cultures, while electron microscopy confirmed the CM particles within ASCs.

**Results:**

This study revealed that ASCs of the omentum of dogs with distemper disease can be infected with CM, indicating their possible involvement in this virus latency and persistence. This suggests that its detection should be considered within the quality control process of stem cells intended for regenerative medicine.

**Conclusion:**

To the best of our knowledge, this is the first study that demonstrates that omentum ASCs from dogs with distemper disease can be infected with CM and may be involved in viral latency or persistence. Our study also suggests that the detection of CM should be considered within the quality control process of stem cells intended for regenerative medicine.

## Introduction

Viral latency is a metastable and unproductive state of infection that may be subsequently reactivated and the cycle of infection repeated. Latent viral infections are associated with several pathologies, such as cancer, congenital defects, neuropathy, cardiovascular disease, chronic inflammation, and immune dysfunctions. The mechanisms controlling the initiation, continuation, and reactivation of latency are complex. They also differ among viral families, species, and strains. Eradication of a latent virus has become a significant, albeit elusive, challenge and requires a more in-depth understanding of the mechanisms that control the viral processes [1]. Some ribonucleic acid (RNA) viruses can induce persistent infection in the host. This occasionally leads to reactive or chronic disease. Some viruses developed this strategy through specific mechanisms that generate persistent infection in some individuals within a population who become reservoirs for the virus [2]. Latency usually occurs in cells that do not allow gene expression of the lytic cycle [3]. Gene expression of latent genes requires cellular and tissue microenvironments that are exceptionally permissive [4]. The immune system cannot detect this because there is no antigen expression in cells infected with viral particles that have entered a latent state [5]. In many cases, such as with cytomegalovirus and other viruses, this latent state hinders the identification of the type of host cell [6]. Cellular culture models that represent latent infection provide valuable information on the processes within the host that regulates the virus’ settling establishment and the continuation of latency, such as that seen with the herpes virus. These models can be used to study the latency in the primary neuronal cells of rats treated with nerve growth factor (NGF), such as that seen with the herpes virus. These models can be used to study the latency in the primary neuronal cells of rats treated with NGF [7–9].

On the other hand, stem cells are multipotent and many persist in several mammalian tissues throughout their lifetime, replacing cells lost during homeostatic renewal, injury, and disease. However, advancing age decreases stem cell function in several tissues, including the prosencephalon [10–12]. Most of these cells remain in a quiescent state, dividing themselves intermittently to maintain tissue homeostasis in mammalian tissues [13]. This can enable virus latency or persistence in some cases. The quiescence displayed by stem cells makes them prone to infection by agents that undergo latency or persistence, such as viruses. Decreased stem cell function contributes to regenerative tissue degeneration, dysfunction, and aging [14]. In this regard, it can be inferred that infection of these cells by a virus that later enters a latent state could accelerate cellular aging through cellular dysfunction, resulting in neurodegenerative diseases. Stem cells are currently being used to treat several pathologies in veterinary medicine. Therefore, their role as target cells for determining the presence of latent viruses is crucial to preventing viral transmission. The canine morbillivirus (CM) is one the most lethal infectious agents in carnivores [15]. It causes distemper disease in dogs. Globally, distemper disease is one of the most important diseases in this species because of its lethality and high infectivity [16, 17]. Moreover, since the neurological disease is associated with viral latency and persistence [18, 19], it is necessary to identify the cells in which it persists after infection.

Based on what is known about viral latency and multipotent stem cell biology, we propose that stem cells may be suitable host cells for CM. Therefore, this study aimed to determine whether adipose stem cells (ASCs) from the lesser omentum of dogs with clinical distemper disease may be infected with CM.

## Materials and Methods

### Ethical approval

All procedures were approved by the ethics committee of the Universidad Científica del Sur, Lima, Peru (approval no. 68-CIEI-AB-CIENTIFICA-2019).

### Study period and location

The study was carried out from January 2020 to December 2021. Molecular experiments were done in the Laboratory of Biochemistry and Molecular Genetics-Universidad Científica del Sur and cell culture experiments were done in the Laboratory of Stem cells-Clinica Veterinaria Enciso.

### Study samples

Different samples were taken from twelve dogs of different breeds between 1 and 10 years of age that were clinically diagnosed with neurological clinical canine distemper (CD). These dogs also tested positive for CM antigens using chromatographic tests in different veterinary clinics and were included in this study. Dogs that had previously been immunized were excluded from the study. Dog owners provided informed consent for sample collection. This study used a descriptive, cross-sectional study design and a convenience non-probability sampling method.

The dogs were anesthetized using a 0.2 mg/kg intramuscular dose of xylazine/ketamine (Agrovet Market, Peru) and a 2 mg/kg intramuscular dose of tramadol [20] to obtain the following samples: Adipose tissue (1 g), cerebrospinal fluid [CSF] (1 mL), urine (3 mL), and blood (3 mL).

### Determination of infection by CM

After the dogs were selected, immunofluorescence was used to determine the presence of CM. Reverse transcription polymerase chain reaction (RT-PCR) of CSF, urine, and blood was conducted at the Veterinary Clinic Laboratory. A polyclonal antibody against CD virus (CDV) (VMRD Inc., Pullman, WA 99163, USA) was used for the immunofluorescence technique. A pair of primers: 5’-ACA GGA TTG TTG CTG AGG ACC TAT-3’ (forward) and 5’-CAA GAT AAC CAT GTA CGG TGC-3’ (reverse) were used to amplify a segment of approximately 290 base pairs (bp) of the CDV nucleoprotein gene by RT-PCR.

### ASCs infected with CM

#### ASC isolation

Adipose tissue samples were taken from the lesser omentum and immediately placed in Dulbecco’s Modified Eagle’s Medium (DMEM) (Gibco) cell culture medium with 1% penicillin/streptomycin/amphotericin (Gibco), then sent to the laboratory for ASC isolation.

The protocol of Enciso *et al*. [21] was followed to isolate ASCs. In brief, the sample was subjected to mechanical and enzymatic disaggregation, then centrifuged, and the supernatant discarded. The fraction containing the mesenchymal stem cells obtained was cultured in culture media bottles under cell culture conditions. Once the culture attained 70% confluence, the cells were detached to be passaged and subcultured in a different bottle. When the second passage was obtained, the cells were cryopreserved at −80°C until use.

Moreover, to confirm that the cells were in fact, mesenchymal cells, they were differentiated into adipogenic, chondrogenic, and osteogenic tissues according to the protocol of Enciso *et al*. [22]. CM-free canine ASCs that were previously cryofrozen in the stem cell bank of the laboratory at the veterinary clinic were used as negative control cells. CM-free ASCs that were infected with the Onderstepoort attenuated viral strain from a commercial vaccine (“Nobivac^®^ Puppy DP,” Intervet International B.V., BOOXMEER- Holland) was used as positive control cells.

### Virus detection

#### Immunofluorescence

This was used to determine the presence of the virus at a cellular level, in accordance with Agallou *et al*. [23] protocol in which cells from the first passage are cultured in sterile coverslips. The ASCs were then recovered and fixed in acetone at room temperature (20°C) for 30 min. They were subsequently incubated in a humidity chamber with 1:1000 polyclonal antibody against CM conjugated with fluorescein isothiocyanate (FITC) (CJ-F-CDV-10M. VMRD, Inc.) at 37°C for 1 h. After adding buffer at a pH of 7.0, the ASCs were incubated further for 10 min with 4’,6-diamidino-2-phenylindole at a concentration of 1 in 20,000 at 20°C. The samples were then washed several times using phosphate-buffered saline (PBS). Finally, a mounting solution was added to read them under a fluorescence microscope (Nikon, Japan) [23, 24].

#### Flow cytometry

To corroborate both the presence and population of CM-infected ASCs, the second-passage cells were frozen and passed through an intracellular dye in accordance with the modified intracellular dye protocol described by Foster *et al*. [25] and Amidzadeh *et al*. [26]. In brief, the cells were washed three times with BPS followed by centrifugation at 500 g for 10 min each time. They were then fixed for 15 min using PBS/PFA 2% at 20°C, then recentrifuged at 500 g for 10 min, and then washed three more times with PBS. Subsequently, 200 μL of PBS/Tween 20 at 0.2% were added to permeate the cells for 30 min. The cells were then centrifuged at 500 g for 10 min and washed three more times with PBS. Finally, the cells were incubated with antibodies against CDV-FITC conjugate (VMRD, Inc., Pullman) for 30 min at 4°C and then washed with PBS. The Guava easyCyte™ (Merck/Millipore, Germany) was used to perform flow cytometry. FlowJo V10 (Applied Cytometry, UK) was used to analyze the results.

#### RT-PCR

RT-PCR was used to detect the presence of viral RNA within the isolated stem cells. The innuPREP Virus DNA/RNA Kit^®^ (Jena, Germany) was used to isolate the CM viral RNA, according to the manufacturer’s protocol. A Qubit fluorometer (Invitrogen™, USA) was used to conduct RNA quantification in accordance with the manufacturer’s protocol.

Frisk *et al*. [27] protocol and primers were used to perform the molecular RT-PCR diagnosis. The RNA obtained was used for cDNA synthesis using the following components and concentrations: RNA = 1 ng–2 μg/rxn, random primers = 0.5 μM, dNTP = 500 μM, and H_2_O. These were introduced into a thermocycler at 65°C for 5 min and 4°C for 1 min. The following components were then added at their respective concentrations: Buffer = 1X, ribosome inhibitor = 20 U/rxn, and OneScript = 200 U/rxn. They were placed in the thermocycler at 94°C for 60 s for initial denaturing, 40 cycles of the denaturing (94°C, 60 s), annealing (60°C, 120 s), and extending (72°C, 120 s) stages, and finally, 72°C for 10 min for the final extension stage.

The following primers were sequenced during the RT-PCR reaction: 5’-ACA GGA TTG CTG AGG ACC TAT-3’ (forward) and 5’-CAA GAT AAC CAT GTA CGG TGC-3’ (reverse). They were used to amplify the CDV nucleoprotein (NP) gene, and these primers allow the amplification of a 290 bp DNA fragment of the nucleoprotein viral gene. The PCR protocol consists of a denaturation period at 94°C for 3 min, followed by 35 cycles involving a denaturation period at 94°C for 30 s, annealing periods at 59.5°C for 30 s, and an extension period at 72°C for 30 s. The last stage is a final extension period at 72°C for 5 min.

A 2% agarose gel was used for the electrophoresis at 110 V using the 100 bp Plus Opti-DNA Marker^®^. After electrophoresis, the gel was stained with ethidium bromide (0.5 μg/mL), incubated for 30 min, and subsequently visualized in an ultraviolet transilluminator and photographed.

#### Transmission electron microscopy (TEM)

The cells presenting the ASCs phenotype in the TEM were cultured in DMEM (Sigma) containing 10% fetal bovine serum and 1% penicillin/streptomycin. Cultures were resuspended and concentrated through centrifugation in microcentrifuge tubes, in which they were fixed, washed, and post-fixed using a solution containing 2.5% glutaraldehyde in 0.1 M phosphate buffer (pH 7.2) for 30 min. The excess fixative agent was removed by washing with phosphate buffer; then the cells were post-fixed using 1% OsO_4_ in double-distilled water for 1 h in an extraction chamber. They were then dehydrated in alcohol solutions in ascending grades – from 30% up to absolute ethanol – finishing with two acetone steps. For the epoxy resin infiltration, they were embedded overnight in a mixture of acetone and Epón-Araldite resin (1:1). They were in acetone/resin (1:2) for 2 h and in pure resin for 2 h (two changes). The tubes were centrifuged for 3 min before the recommended resting times for each step were over. The infiltrated samples were polymerized on an electric stove at 60°C.

The blocks obtained were carved and cut using a glass knife ultramicrotome. Ultrathin sections were collected in 300 mesh copper grids. In accordance with Reynolds [28], the sections were impregnated with uranyl acetate and lead citrate. These cuts were analyzed and photographed in a TEM (model: Tecnai; brand: FEI).

## Results

### ASCs infected with CM

**Figure-1:**
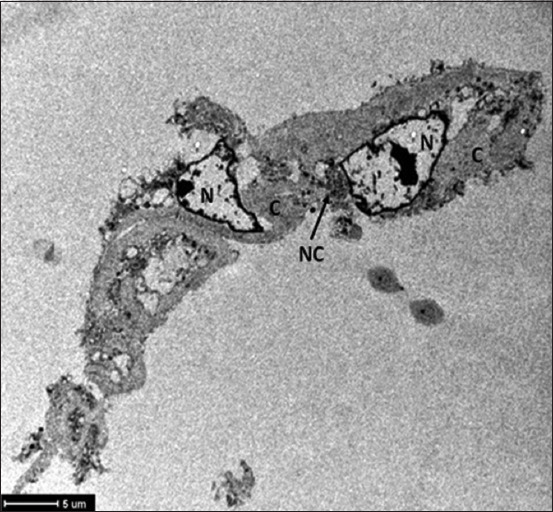
Transmission electron microscopy of adipose stem cell infected with canine distemper virus. N=Nucleus, C=Cytoplasmic matrix, NC=Viral nucleocapsid.

ASCs were observed using an inverted microscope. They showed adhesion to plastic and an elongated morphology, similar to fibroblasts, which was ratified by TEM (Figure-1). The cytopathic effect that has been described in cellular cultures of this virus was not observed in this study.

The multipotentiality of the isolate was shown by differentiation into three consensual lineages: Adipogenic, chondrogenic, and osteogenic. Oil Red O dye was used to identify adipocytes, Alcian Blue dye for osteoblasts, and Alizarin Red S (which reacts with mineralized material) for osteocytes. These attributes have been described as inherent in adult stem cells [29]. The presence of CM was confirmed after analyzing the ASC isolates.

### Virus detection

#### Immunofluorescence

**Figure-2:**
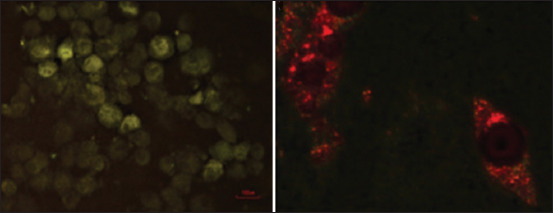
(a) Adipose stem cells (ASCs) infected with the vaccine canine distemper virus (40×). (b) ASCs from dogs with a natural canine distemper virus infection (100×).

**Figure-3:**
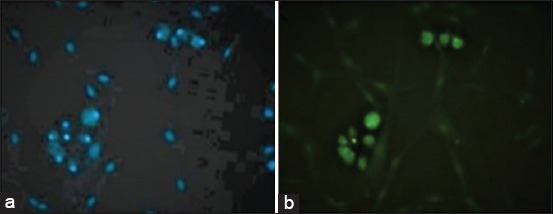
Adipose stem cells from dogs naturally infected with canine morbillivirus. (a) 4’,6-Diamidino-2-phenylindole dye and (b) direct immunofluorescence using anti-canine distemper virus antibody (40×).

The presence of CDV was confirmed by direct immunofluorescence (DIF) using a CDV-specific antibody on the first-passage ASCs from dogs with clinical distemper after culturing for 72 h (Figures-2b, 3a, and 3b). Positive control cells containing vaccine CDV also showed the presence of the virus (Figure-2a). In contrast, the uninfected ASCs that were used as negative controls showed no fluorescent reaction.

#### Flow cytometry

**Figure-4:**
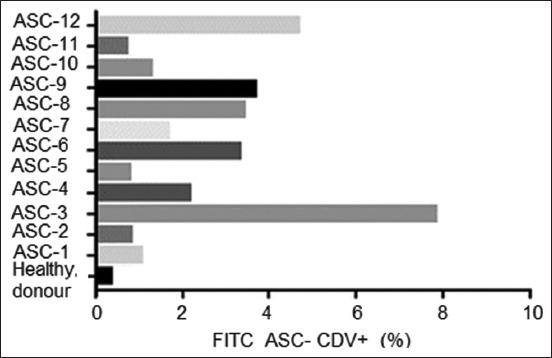
Percentage of canine distemper virus-positive adipose stem cells in isolates obtained from the dogs studied.

**Figure-5:**
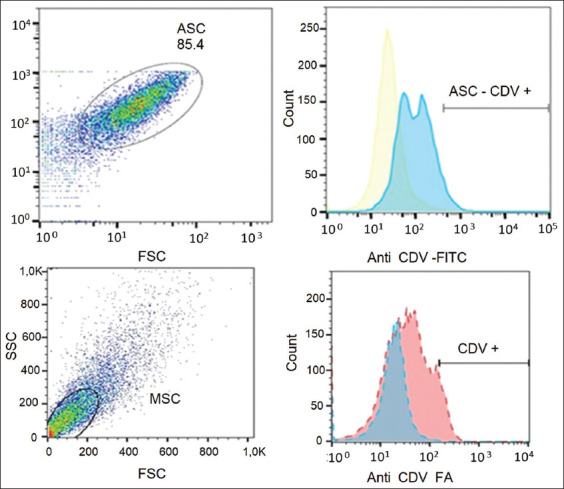
Flow cytometry. Selection protocol using stem cells from healthy subjects as a blank (blue curve) versus stem cells from sick subjects (red curve).

Twelve second-passage ASC isolates from dogs with clinical CD were analyzed using anti-CDV-FITC antibodies. CM-free canine ASCs were used as negative controls. The 12 isolates exhibited different cell populations, ranging from >2% to 8%, with samples 3 and 12 presenting the largest CDV-positive cell population, whereas the negative control from a healthy dog showed <1% (Figures-4 and 5).

#### RT-PCR

**Figure-6:**
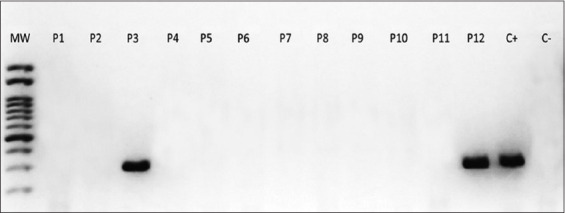
Reverse transcription polymerase chain reaction of adipose stem cells from dogs infected with canine distemper virus. P3 and P12: Positive samples displaying similar bands as C+. C+: Positive control. C−: Negative control. MW=Molecular weight marker.

RT-PCR was used to detect CM following the protocol described in the “Materials and Methods” section. The presence of the virus was established in two ASC samples (samples 3 and 12) isolated from dogs with distemper disease (Figure-6).

#### TEM

**Figure-7:**
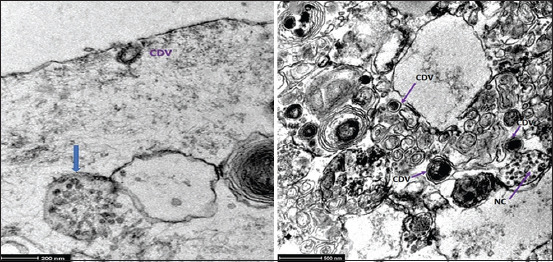
Transmission electron microscopy of adipose stem cell infected with canine distemper virus. (a) Intracytoplasmic nucleocapsid (arrow). Canine distemper virus: Virion in the cell periphery. (b) Cytoplasmic inclusion with viral nucleocapsids.

**Figure-8:**
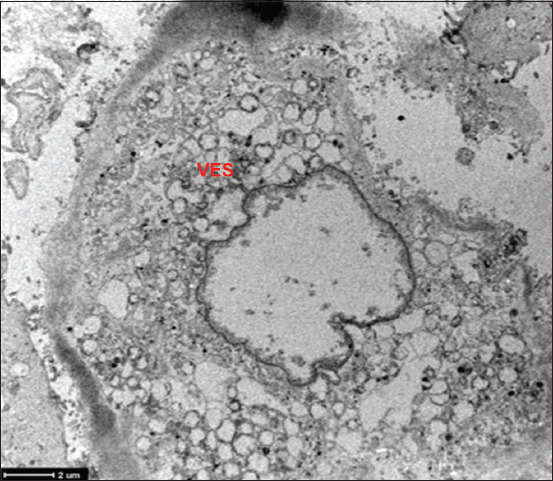
Transmission electron microscopy of adipose stem cell infected with canine distemper virus. Nucleus with peripheral chromatin and nuclear matrix lysis. VES=Double-membrane vesicle clusters in the cytoplasm.

The use of TEM demonstrated the presence of virions within the peripheral cytoplasm, as well as viral nucleocapsid clusters (Figure-7a) in ASCs obtained from the omentum of dogs showing clinical signs of distemper disease. The infected cells containing several viral particles in their cytoplasm had large groups of lamellar bodies and cytoplasmic inclusions with viral nucleocapsids and vacuolization (Figure-7b). However, at a nuclear level, nuclei with peripheral chromatin and nuclear matrix lysis, and marginal chromatin were observed (Figure-8).

## Discussion

Cytometric evidence of viral particles demonstrated for the 1^st^ time that CM, which can cause a systemic infection of multiple organs and tissues, infects ASCs in dogs with neurologic distemper disease. RT-PCR showed viral genes and TEM showed mature viral particles.

This demonstrates that ASCs are susceptible to infection with CM. However, this highlights that the cells infected with CM did not show any evidence of the cytopathic effect as described by Sultan *et al*. [30]. Nevertheless, TEM showed changes in their organelles.

In that regard, although it was not the purpose of this study, we consider that ASCs may not have any of the receptors recognized by the virus, including the SLAM receptor – which is ** found in thymocytes, activated lymphocytes T and B, dendritic cells, and macrophages – the Nectin-4 receptor, which is found in epithelial cells [31–33], or a potential third receptor, as in what may be present in astrocytes which do not display the aforementioned receptors [34].

The CM is a single-stranded negative-sense RNA virus [35] that can infect multiple cells in dogs. It has an affinity for circulating epithelial and nerve cells [36]. ASCs, however, are immature and their susceptibility to this virus has not been demonstrated. Therefore, the results of this study contrast recent proposals by other researchers who showed that stem cells protect themselves from RNA viruses by expressing an isoform of the Dicer enzyme known as aviD. This enzyme enhances RNA viral interference, thereby limiting the infection of mammalian stem cells by RNA viruses [37].

On the other side, RT-PCR, in our study, showed that two of the 12 second-passage isolates obtained from the omentum of dogs with neurological distemper had high percentages of viral particles. The remaining isolates showed negative RT-PCR results for CM, despite all the animals having a positive CDV rapid test. This may suggest that the disease stage of the animals did not involve adipose tissue yet.

According to these results, we can hypothesize that the CM found within the ASCs may be latent. This would explain the absence of a cytopathic effect secondary to silencing the lysis-related genes [38]. This hypothesis is based on what has been described for the human immunodeficiency virus (HIV), in which the low-oxygen environment within the lymph nodes suppresses HIV replication and promotes latency [39]. Another study demonstrated that adipose tissue in mice has oxygen concentrations of around 3% [40]. In addition, another study described mesenchymal stem cell involvement in the reactivation of latent HIV [41].

The presence of a mechanism of viral persistence was proposed years ago. Although this possible mechanism is yet to be molecularly explained, it would allow evasion of the central nervous system without the need to release large amounts of viral particles [42]. Meanwhile, persistent infection with the release of viral particles up to 17 months post-primary infection has been described [43]. In contrast, the Zika virus has been described to reduce organoid growth derived from embryonic stem cells, leading to apoptosis of these cells [44]. Moreover, cross-communication between viruses and stem cells through miRNAs has been demonstrated [45] and may be useful for exploring therapeutic approaches to control viral infections and understanding stem cell latency and reactivation mechanisms [46].

## Conclusion

This study showed that the ASCs of dogs with clinical neurological phase distemper disease are infected with the CM. However, there is no evident cytopathic effect in the cell culture. This suggests their possible involvement in the latency of this virus, and that ASCs used for regenerative therapy should undergo screening for this virus as part of their quality control. A limitation of the execution of this work was the difficulty in obtaining a larger number of samples from veterinary clinics. Based on these results, the mechanism by which the virus enters and persists in the host cell needs to be studied.

## Authors’ Contributions

JE and NE: Conception and designed the study. NR and FA: Methodology and investigation. JEB: Supervised the study and analyzed and validated the data. JMI, MF, NE, and JE: Drafted, edited, and revised the manuscript. All authors have read and approved the final manuscript.
